# Mid-arm circumference and mid-arm/head circumference ratio in term newborns

**DOI:** 10.1590/S1516-31802004000200004

**Published:** 2004-03-01

**Authors:** Bettina Barbosa Duque Figueira, Conceição Aparecida de Mattos Segre

**Keywords:** Cephalometry, Anthropometry, Fetal growth restriction, Newborn, Perímetro braquial, Medição do perímetro braquial, Cefalometria, Antropometria, Retardo do crescimento fetal, Recém-nascido

## Abstract

**CONTEXT:**

Mid-arm circumference of the newborn is strongly associated with birth weight and is a very good indicator of low and insufficient birth weight. However, there are few Brazilian studies on the relationship between mid-arm and head circumferences and, thus, this does not form part of the routine evaluation for newborns.

**OBJECTIVES:**

To establish the mid-arm circumference and mid-arm/head circumference ratio in a population of term newborns.

**TYPE OF STUDY:**

Cross-sectional study carried out between June 1997 and August 1999.

**SETTING:**

Hospital Maternidade Leonor Mendes de Barros, São Paulo.

**PARTICIPANTS:**

Term newborns (66 males and 65 females) of appropriate growth for gestational age, whose mothers were healthy, were included in the study.

**MAIN MEASUREMENTS:**

Arm circumference, arm circumference/head circumference ratio, birth weight and gestational age were measured within 48 hours of birth. Data were considered significant when p < 0.01.

**RESULTS:**

The mean values for the mid-arm circumference were 10.76 cm (standard deviation, SD = 0.68) for females and 10.76 (SD = 0.81) for males. The mean value for the mid-arm/head circumference ratio was 0.31 (SD = 0.02) for both sexes. Mid-arm circumference values were significantly related to birth weight and gestational age, whereas mid-arm/head circumference ratio was related only to birth weight.

**CONCLUSIONS:**

Mid-arm circumference and mid- arm/head circumference ratio values were established for the studied population. It was possible to obtain curves for both mid-arm circumference and mid-arm/head circumference ratio in relation to birth weight. However, for mid-arm circumference, it was only possible to obtain curves in relation to gestational age. The use of the regression curves did not seem powerful enough to predict the mid-arm circumference and mid-arm/head circumference ratio in this population of term newborns. There were no gender differences for either of the measurements studied.

## RESUMO

**CONTEXTO:**

O perímetro braquial do recém-nascido está fortemente relacionado ao peso de nascimento e constitui bom indicador de baixo peso e de peso insatisfatório ao nascer. No entanto, há carência de estudos nacionais que forneçam informações acerca da relação perímetro braquial/perímetro cefálico no período neonatal, de modo que essa medida não faz parte da rotina de avaliação do recém-nascido.

**OBJETIVOS:**

Estudar a medida do perímetro braquial e a relação perímetro braquial/perí- metro cefálico, em uma população de recém-nascidos de termo.

**TIPO DE ESTUDO:**

Estudo de corte transversal, realizado entre junho de 1997 e agosto de 1999.

**LOCAL:**

Hospital Maternidade Leonor Mendes de Barros. São Paulo e Instituto de Assistência Médica ao Servidor Estadual de São Paulo (IAMSP — SP)

**PARTICIPANTES:**

Foram incluídos no estudo 131 recém-nascidos: 66 do sexo masculino e 65 do feminino.

**PROCEDIMENTOS:**

Foram incluídos recém- nascidos de mães sadias, no termo, de gestação única, adequados para a idade gestacional, sem malformações graves ou alterações clínicas sugestivas de restrição de crescimento intra-uterino. Os recém-nascidos foram medidos nas primeiras 48 horas de vida e os dados, analisados mediante um nível de significância de 1%.

**VARIÁVEIS ESTUDADAS:**

Perímetro braquial, relação perímetro braquial/perímetro cefálico, peso ao nascer, idade gestacional, sexo.

**RESULTADOS:**

Foram incluídos no estudo 131 recém-nascidos: 66 do sexo masculino e 65 do feminino. O perímetro braquial apresentou valor médio de 10,76 ± 0,68 no sexo feminino e 10,76 ± 0,81 no sexo masculino. A relação perímetro braquial/perímetro cefálico apresentou valor médio de 0,31 ± 0,02 para ambos os sexos. Não se observou diferença significativa em nenhuma das medidas em relação ao sexo. Os valores do perímetro braquial apresentaram correlação positiva com o peso de nascimento e com a idade gestacional ao passo que a relação perímetro braquial/perímetro cefálico relacionou-se apenas com o peso ao nascer. Foram obtidas curvas de regressão para o perímetro braquial em relação ao peso e à idade gestacional, e para a relação perímetro braquial/perímetro cefálico em relação ao peso de nascimento, apenas. A associação entre o perímetro braquial e a idade gestacional foi melhor representada através de regressão quadrática.

**CONCLUSÕES:**

Foram estabelecidos valores de perímetro braquial e da relação perímetro braquial/perímetro cefálico na população estudada. Foi possível obter curvas de regressão para o perímetro braquial em relação tanto ao peso de nascimento quanto à idade gestacional. No entanto, para a relação perímetro braquial/perímetro cefálico, foi possível obter curva de regressão linear apenas em relação ao peso de nascimento. Não se demonstrou diferença entre os sexos para nenhuma das medidas estudadas. Os baixos coeficientes de correlação encontrados, bem como o comportamento das medidas em relação à idade gestacional, sugerem que a utilização de curvas de regressão linear não parece um bom método para predizer os valores do perímetro braquial ou da relação entre o perímetro braquial e o perímetro cefálico, em recém-nascidos de termo.

## INTRODUCTION

Early prediction of the risks to which a child is exposed at birth allows for better organization of perinatal care and optimization of available resources, thus avoiding unnecessary controls and assuring maximum attention for those children who really need it.^1^ With this objective in mind, several indicators have been recommended. Among these, the birth weight in relation to gestational age has frequently been used for classifying newborns according to the intrauterine growth experienced. The three categories are normal intrauterine growth (AGA, or appropriate for gestational age), subnormal growth (SGA, or small for gestational age) or supranormal growth (LGA, or large for gestational age).^2^ Several criteria have been used for separating these three categories, the most common being based on percentiles from weight for gestational age distribution in a reference population.^3^

Several studies have led to the conclusion that the newborn's nutritional status is more important than birth weight alone for identifying perinatal risks.^4,5^ Perinatal risk assessment by weight percentile criteria has been shown to be insufficient, thus requiring the determination of additional or alternative indices to improve this evaluation. The mid-arm circumference (MAC) measurement is less affected by subclinical edema than weight alone and is relatively easy to obtain. This has led several authors to employ it as an important tool for identifying malnutrition and mortality risk.^6-9^ However, most of the nutritional studies involving anthropometric parameters have used the weight/height ratio and its derivatives – body mass index and ponderal index^7,10^ – in order to evaluate individual body proportionality.

With the aim of evaluating preschool children's nutritional condition, Kanawati and McLaren^11^ were the first authors to propose the mid-arm circumference/head circumference (MAC/HC) ratio for such an evaluation. This ratio is easily obtained by simple and non-expensive equipment, with minimal training requirements. Its use in the neonatal period was introduced in 1986, when Sasanow et al.^12^ established reference values for newborns of gestational age 25 to 42 weeks with appropriate growth for gestational age.

In Brazil, Dias^13^ and Alves et al.^14^ showed that the newborn's mid-arm circumference was strongly related to birth weight, thus representing a good marker for low and very low birth weight. However, a large number of neonatologists are not aware of the potential usefulness of such measurements, with the result that these anthropometric parameters are remarkably underused in Brazil.

The objectives of the present study were the following:

- to establish mid-arm circumference values and the mid-arm circumference/head circumference ratio among a population of term Brazilian newborns, according to gestational age and birth weight;
- to investigate the occasional differences in anthropometric variables found in the present study according to gender;
- to evaluate the possibility of obtaining correlation curves for the studied variables according to gestational age and birth weight.

## METHODS

A cross-sectional study was performed among term live birth newborns, from June 1997 to August 1999, at Hospital Maternidade Leonor Mendes de Barros, São Paulo, Brazil, a public maternity hospital within the healthcare system that serves a low-income population and is used as a reference center for high-risk pregnancies.

### Inclusion and exclusion criteria

The study group consisted of newborns from single pregnancies, with gestational ages of between 37 weeks and 41 weeks and 6 days, as estimated by Capurro's method,^15^ who were classified as appropriate for gestational age via the weight/gestational age (W-GA) criterion. These newborns were examined by the main author within their first 48 hours of life. Only newborns whose mothers agreed to participate in the study were included.

Newborns whose mothers presented complications during pregnancy, such as previous or pregnancy-related arterial hypertension, infection, previous or pregnancy-related diabetes, or had a history of illegal drug abuse or smoking habits, if more than 10 cigarettes per day, were excluded. Newborns with major malformations, hydropic appearance or presenting signs of intrauterine growth restriction such as an Apgar score of less than 7 in the fifth minute of life, hypoglycemia, hypocalcemia or polycythemia and those whose mothers denied authorization were also excluded.

Thus, the total sample included in the study comprised 131 newborns.

### Gestational age determination

In order to determine the gestational age, a daily service neonatologist or a duly trained pediatric resident examined the children 6 to 12 hours after birth. The "term" concept was applied to those newborns whose gestational ages ranged from 37 weeks to less than 42 completed weeks: from 259 to 293 gestational days, as calculated by Naegele's rule.^15^ The gestational age was also estimated via clinical- neurological examination of the newborn (using Capurro's method)^15^ and was expressed as "completed weeks".^16^

### Intrauterine growth adequacy determination

For this purpose, the weight-gestational age criterion was applied, considering the 10^th^ percentile as the lower limit for newborns to be appropriate for gestational age, at the border with small for gestational age, and the 90^th^ percentile as the upper limit for newborns to be appropriate for gestational age, at the border with large for gestational age. The newborns were classified according to the weight-gestational age curve routinely used in the hospital where the study was carried out.^17^

### Anthropometric measurements:

#### Birth weight

The weight was obtained with the naked infant in dorsal decubitus, soon after birth, still in the delivery room, using an electronic balance with a maximum capacity of 15 kg and a minimum of 125 g, and 5 g subdivisions, previously calibrated by the Brazilian National Institute of Weights and Measures (Inmetro). The measurements were taken by an attending nurse or the neonatologist attending the delivery room.

#### Circumferences

**Figure 1:**
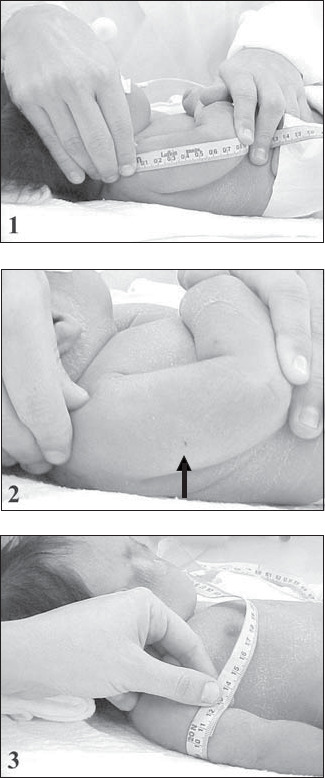
Mid-arm circumference: obtaining the measurement. 1. length of the arm; 2. mid-arm point (arrow); 3. measurement of circumference at the mid-arm point.

All measurements were taken by the main author or the neonatology resident, according to the technique previously described,^12^ so that this could not represent an impediment in comparing the final results. The arm and head circumferences were measured within the first 48 hours of life, using a fiberglass non-extendable measuring tape, with a width of 1.0 cm and subdivisions of 0.1 cm. The mid-arm circumference was obtained from the left arm, at the mid point between the acromion and olecranon, with the newborn in dorsal decubitus with the arm lying laterally to the trunk. The midpoint was located by measuring the distance between the acromion and olecranon extremities, with the elbow flexed at an angle of 90°. A small mark was made at the identified point (Figure 1). A total of three consecutive measurements were taken for each newborn, and the mean value (rounded to the nearest 0.1 cm) was considered for analysis.

The head circumference was measured with the newborn in dorsal decubitus. The measuring tape was placed along the occipital-frontal circumference, just over the eyebrows and the occiput, in order to obtain the largest measurement. The maximum value of three consecutive measurements was considered, rounded to the nearest 0.1 cm.^18^

### Statistical analysis

The data processing was done using the Statistical Package for the Social Sciences (SPSS) version 9.0 software. The probability level p < 0.01 was considered to be significant. Statistical analyses were performed to estimate the arithmetic mean and standard deviation, followed by the Kolmogorov-Smirnov test to determine the normal distribution of the variables studied: arm circumference and arm circumference/head circumference ratio. The Student t test was used to compare genders, and a correlation matrix was built in order to test associations with gestational age and birth weight among the studied variables. Linear regression was applied considering birth weight as an independent variable. Multiple regression analyses were used, in which gestational age (GA), gestational age squared (GA^2^) and gestational age cubed (GA^3^) were considered as independent variables.

The present study was duly approved by the Research Ethics Committee of Hospital Maternidade Leonor Mendes de Barros.

## RESULTS

The study comprised 131 newborns: 66 males and 65 females. The mean birth weight of the sample was 3,177 g, ranging from 2,330 g to 3,910 g. The average gestational age was 39 complete weeks, ranging from a minimum of 37 weeks to a maximum of 41 weeks.

Table 1 shows the mean and standard deviations for male and female newborns. The normality test (Kolmogorov-Smirnov) showed that both variables studied followed the normal distribution. The Student t test, used in order to identify possible differences between sexes, showed no significant differences for any of the evaluated parameters, and therefore the sample was considered as a whole.

**Table 1:** Patient characteristics — mean values and standard deviation by gender

Characteristics	Gender		Calculated "t"	significance (p)
	Female	Male		
Age (hours)	28.52 ± 11.68	29.18 ± 11.08	0.34	0.737 (NS)
Birth weight (g)	3154.54 ± 296.82	3200.20 ± 311.98	0.86	0.393 (NS)
Gestational age (weeks)	39.15 ± 1.11	39.26 ± 1.06	0.55	0.845 (NS)
Arm circumference (cm)	10.76 ± 0.68	10.76 ± 0.81	0.04	0.965 (NS)
AC/HC	0.31 ± 0.02	0.31 ± 0.02	1.41	0.160 (NS)

*NS = Not significant; AC/HC = arm circumference/head circumference.*

**Figure 2:**
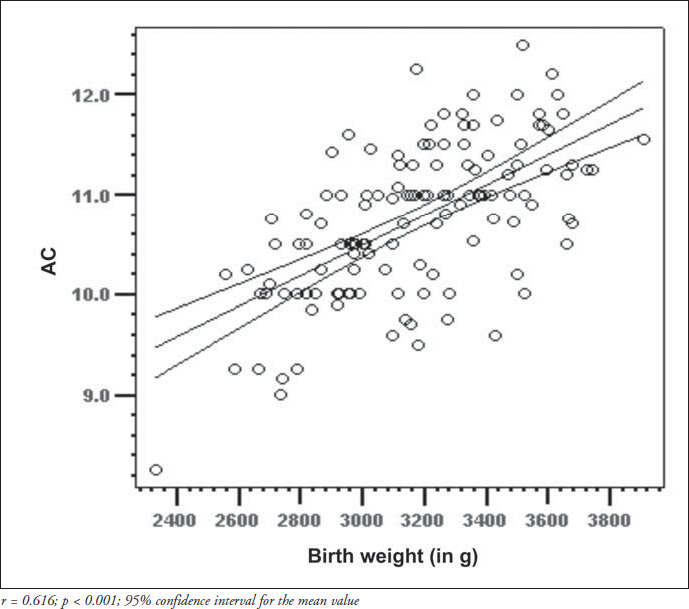
Regression line corresponding to the mid-arm circumference (AC) of newborns with gestational ages between 37 and 41 completed weeks, in relation to birth weight.

**Figure 3:**
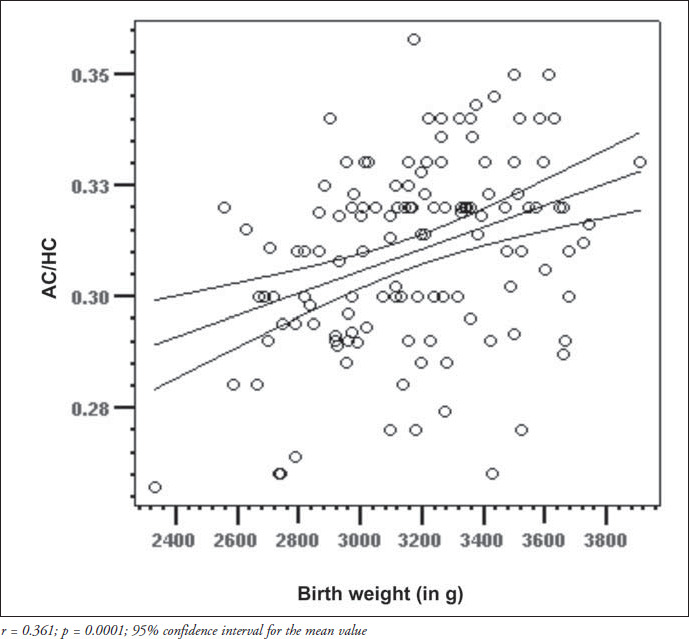
Regression line corresponding to the arm circumference/head circumference ratio (AC/HC) of newborns with gestational ages between 37 and 41 completed weeks, in relation to birth weight.

**Figure 4:**
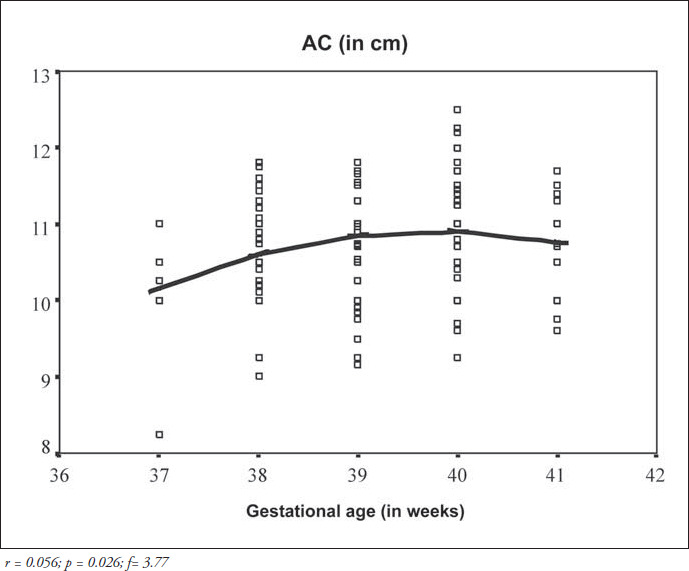
Quadratic regression line corresponding to the arm circumference (AC) of newborns with gestational ages between 37 and 41 completed weeks, in relation to gestational age.

The correlation matrix for the parameters of the overall group is shown in Table 2. Birth weight was positively correlated with both arm circumference and arm/head circumference ratio (p < 0.0001 for both), whereas the correlation coefficients between gestational age and arm circumference or arm/head circumference ratio were low and not significant. The quadratic regression for arm circumference proved to be significant (r^2^ = 0.056; f = 3.77; p = 0.026), thereby showing that the best adjustment for the curve could be obtained by using the squared gestational age (Figure 4). For the mid-arm circumference/head circumference ratio, the regression analysis did not prove to be significant (r^2^ = 0.0028; f = 1.86; p = 0.159). It was possible to obtain linear equations and their graphs using quadratic regression (Figure 4), including the individual confidence interval calculations for the midarm circumference and mid-arm circumfer- ence/head circumference ratio versus birth weight (Figures 2 and 3), and for the midarm circumference versus gestational age. Although significant, the regression coefficient (r^2^) for mid-arm circumference over gestational age was low and, consequently, the accuracy of estimating arm circumference by gestational age was not recommended.

**Table 2:** Correlation matrix between the various parameters of all the 131 newborns*

	Weight	AC	Gestational age	AC/HC
**Weight**				
**AC**	0.616			
	p = 0.000			
**Gestational Age**	0.335	0.168		
	p = 0.000	p = 0.056		
**AC/HC**	0.361	0.876	0.076	
	p = 0.000	p = 0.000	p = 0.391	

* *Person multiple correlation; correlation is significant at the 0.01 level, two-tailed; AC = arm circumference; AC/HC: arm circumference/head circumference.*

It was therefore decided that we should use the result from the quadratic regression as an adjustment factor for arm circumference and then calculate the mean and standard deviation for the adjusted variable, thereby obtaining an arm circumference value independent of gestational age. Thus, the range from the average minus two standard deviations to the average plus two standard deviations, which corresponds to 95% of the distribution, could be used as the parameter for the normal range. The corrected value was obtained as follows:


$$observed\;arm\;circumference+\;b\;\times \text{ (}mean\;gestational\;age-gestational\;age\text{)}+\;c\times \text{ (}mean\;gestational\;age^{2}-\;gestational\;age^{2}\text{),}$$

where: b = regression constant for gestational age and c = regression constant for gestational age^2^. This new distribution presented a mean value of 10.82 cm and standard deviation of 0.73. Thus, 9.36 cm to 12.28 cm could be considered as the normal range, which included 95% of the studied population.

## DISCUSSION

As a general rule, anthropometric measurements present systematic differences between genders. Therefore, whenever the intention is to study such measurements, it is essential to report the gender of the studied population and study any possible differences between sexes.^18^ However, mid-arm circumference seems to show lesser variation related to gender, and the differences were not significant in a study among infants aged 3 months to 4-years by Kanawati et al.^19^ Among studies of the neonatal period, our data also match the findings of the majority of authors, who did not find any difference between genders in relation to the mid-arm circumference and mid-arm circumference/head circumference ratio values.^6,17,20^ However, in a study among newborns of gestational ages ranging from 34 to 42 weeks, Jiménez Garcia et al.^21^ found different mid-arm circumference values according to gender, with male measurements greater than female ones. Our study did not corroborate these findings.

Mid-arm circumference increases as pregnancy progresses and this is mainly due to fat accumulation in the subcutaneous deposits of the upper extremity.^22^ In the present study, mid-arm circumference showed a linear increase in relation to birth weight, agreeing with the literature on this subject,^12,20^ but the correlation with gestational age was low. It was thus hypothesized that the use of the mean value plus or minus two standard deviations, would better predict the normal values in this population.

Significant variation exists in mid-arm circumference values among different populations. These differences in measurements may be due to several factors, including each population's genetic characteristics and nutritional status, as well as possible differences in measurement procedures.^23^ The mid-arm circumference values found in the present study showed lesser dispersion of means and stronger correlation coefficient when considered in relation to birth weight, rather than in relation to gestational age. Our data differ from those described by most of the authors who have already studied this association.^12,20^ However, our findings were similar to those described by Sánchez et al.^24^ in a Chilean population comprised mainly of term newborns. In the Chilean study, in addition to identifying greater scattering of mid-arm circumference values when associated with gestational age, rather than with birth weight, the authors showed that there was progressive increase in those measurements as gestational age progressed and also found slightly lower values for the mid-arm circumference among the 42-week gestational age newborns. Similar findings were also demonstrated for birth weight. This fact matches some authors' descriptions of the reduction in intrauterine growth rate that takes place by the end of the gestational period. Normal newborns tend to present minor variations in their subcutaneous fat content at term,^22,25^ thus resulting in lesser mid-arm circumference variation and accounting for the low correlation coefficients found in this study.

Malnutrition is characterized by a remarkable lack of body proportions and the midarm circumference/head circumference ratio identifies this characteristic, since both arm and head circumferences are affected by malnutrition in different ways. Kanawati and McLaren^11^ demonstrated that, among normal infants and preschool children ranging from 3 months to 4 years old, the mid-arm circumference/head circumference ratio remains practically constant, with a mean value of 0.31. In the neonatal period, however, several authors have demonstrated that this ratio varies directly with gestational age.^12,20,26^ In the present study, the mid-arm circum-ference/head circumference ratio also showed direct correlation with birth weight, but the correlation was not significant regarding gestational age.

Considering that, from a nutritional point of view, mid-arm circumference provides information similar to weight,^26^ it can be expected that the growth in this measurement will present a pattern similar to what is seen for the weight. Therefore, instead of showing indefinitely increasing values as the gestational age progresses, the mid-arm circumference and consequently the mid-arm circumference/head circumference ratio would tend to present a reduction in their growth rate when reaching term, in keeping with weight behavior. In the present study, although only term newborns were analyzed, this behavior is represented by the quadratic regression graph shown in Figure 4, which shows a slowdown after 38 weeks of gestational age and a drop after 40 weeks. Such behavior was also described by Golebiowska et al.^26^ and Balcazar et al.,^27^ in their studies consisting of pre-term and term newborns, and by Ramos^28^ in his paper on ponderal index.

Yau & Chang^25^ studied a Chinese newborn population ranging from 27 to 42 weeks of gestational age in order to obtain reference indices for body proportions. They found that, except for the head circumfer-ence/length ratio, all other indices, including the mid-arm circumference/head circumference ratio, showed a significant correlation with gestational age, when the population was considered as a whole. However, when analyzing these correlation coefficients separately for pre-term and term newborns, the latter group showed no significant variation in the ponderal index and a weaker correlation coefficient of the mid-arm circum- ference/head circumference ratio, in comparison with the pre-term group. These authors found that there was a reduction in the mid-arm circumference/head circumference ratio values after the gestational age of 40 weeks, similar to our findings.

The correlation coefficient found for term newborns in the present study was positively associated with birth weight and, although statistically significant, was a much weaker association than those described in the studies previously mentioned. The reason for these findings may be the characteristics of the population studied, which comprised only term newborns. It is possible that the newborn population evaluated by our group showed similar behavior to the newborns studied in Poland,^26^ with mid-arm circumference/head circumference ratio showing an association with both weight and gestational age until reaching term and after that, only with birth weight. The explanation for this may lie in the mid-arm circumference behavior found in term newborns that was mentioned earlier: these values would be determinant in calculating the mid-arm circumference/head circumference ratio. Such findings may imply the need for differentiated curves and criteria for evaluating pre-term and term newborns via these anthropometric parameters.

Reports including only term newborns do not describe the construction of regression curves for the parameters of the present study, and only determine mean values for the mid-arm circumference and/or mid-arm circumference/head circumference ratio.^29^ Gueri et al.^30^ even mentioned the impossibility of obtaining a linear equation for their cases. In the present study, although we were able to obtain regression equations for the data by using only term infants, the correlation coefficients found here were weaker than those described in the literature. This may suggest that, for term newborns, the use of fixed parameters such as the mean value plus or minus two standard deviations would better fit the behavior of these anthropometric measurements.

## CONCLUSIONS

The present findings showed the direct association of mid-arm circumference with both birth weight and gestational age, in accordance with descriptions presented in the literature. However, this correlation was stronger for birth weight than for gestational age. The mid-arm circumference/head circumference ratio was associated only with birth weight and not with gestational age.

No significant associations were noted between gender and either mid-arm circumference or mid-arm circumference/head circumference ratio.

It was possible to obtain regression curves for the mid-arm circumference, and for the mid-arm circumference/head circumference ratio in relation to birth weight alone. The correlation coefficients in this term newborn population were weaker than those reported in literature for populations including both pre-term and term newborns, thereby resulting in a low degree of predictability for the studied variables. Thus, our findings suggest that the use of curves obtained by linear regressions may not be a reliable way to predict the mid-arm circumference and mid-arm/head circumference ratio in term newborns.

The values described in the present study for a term newborn population will need to be reevaluated with regard to their applicability to other populations.
